# Alternative Complement Pathway Deficiency Ameliorates Chronic Smoke-Induced Functional and Morphological Ocular Injury

**DOI:** 10.1371/journal.pone.0067894

**Published:** 2013-06-25

**Authors:** Alex Woodell, Beth Coughlin, Kannan Kunchithapautham, Sarah Casey, Tucker Williamson, W. Drew Ferrell, Carl Atkinson, Bryan W. Jones, Bärbel Rohrer

**Affiliations:** 1 Division of Research, Department of Neurosciences, Medical University of South Carolina, Charleston, South Carolina, United States of America; 2 Department of Ophthalmology, Medical University of South Carolina, Charleston, South Carolina, United States of America; 3 Department of Microbiology and Immunology, Medical University of South Carolina, Charleston, South Carolina, United States of America; 4 Moran Eye Center, University of Utah, Salt Lake City, Utah, United States of America; 5 Research Service, Ralph H. Johnson VA Medical Center, Charleston, South Carolina, United States of America; Institut de la Vision, France

## Abstract

**Background:**

Age-related macular degeneration (AMD), a complex disease involving genetic variants and environmental insults, is among the leading causes of blindness in Western populations. Genetic and histologic evidence implicate the complement system in AMD pathogenesis; and smoking is the major environmental risk factor associated with increased disease risk. Although previous studies have demonstrated that cigarette smoke exposure (CE) causes retinal pigment epithelium (RPE) defects in mice, and smoking leads to complement activation in patients, it is unknown whether complement activation is causative in the development of CE pathology; and if so, which complement pathway is required.

**Methods:**

Mice were exposed to cigarette smoke or clean, filtered air for 6 months. The effects of CE were analyzed in wildtype (WT) mice or mice without a functional complement alternative pathway (AP; C*FB^−/−^*) using molecular, histological, electrophysiological, and behavioral outcomes.

**Results:**

CE in WT mice exhibited a significant reduction in function of both rods and cones as determined by electroretinography and contrast sensitivity measurements, concomitant with a thinning of the nuclear layers as measured by SD-OCT imaging and histology. Gene expression analyses suggested that alterations in both photoreceptors and RPE/choroid might contribute to the observed loss of function, and visualization of complement C3d deposition implies the RPE/Bruch's membrane (BrM) complex as the target of AP activity. RPE/BrM alterations include an increase in mitochondrial size concomitant with an apical shift in mitochondrial distribution within the RPE and a thickening of BrM. C*FB^−/−^* mice were protected from developing these CE-mediated alterations.

**Conclusions:**

Taken together, these findings provide clear evidence that ocular pathology generated in CE mice is dependent on complement activation and requires the AP. Identifying animal models with RPE/BrM damage and verifying which aspects of pathology are dependent upon complement activation is essential for developing novel complement-based treatment approaches for the treatment of AMD.

## Introduction

Age-related macular degeneration (AMD) is one of the leading causes of blindness in the elderly among Western populations. AMD is a common, late-onset maculopathy that can be diagnosed in one of two forms: atrophic, “dry”; or neovascular, “wet” [1]. The atrophic form of the disease is best characterized by the presence of deposits rich in lipoprotein known as drusen (≥63 µm in size) in the subretinal space between Bruch's membrane (BrM) and the retinal pigment epithelium (RPE) in the macular region of the eye, and may also be associated with pigmentary abnormalities. These alterations can be easily visualized in patients through fundoscopic imaging. The neovascular form is typically associated with proliferation of the choroidal blood vessels through BrM into the subretinal space. These vessels then leak blood and proteins either into the subretinal or sub-RPE space [2], leading to increased photoreceptor cell death. Although patients can be diagnosed with either form of the disease, the atrophic form is the most common, making up 90 percent of all cases [3].

AMD is a complex, multifaceted disease that is influenced by genetic and environmental factors. Several of the main genetic risk factors are polymorphisms occurring in complement genes, including the alternative pathway (AP) inhibitor, CFH (complement factor H [4]–[7]), CFB (complement factor B [8]), C2 (complement component 2 [8]), and C3 (complement component 3 [8], [9]). Several of these complement components have been found to be associated with pathological features of AMD. Drusen present in donor eyes of patients with confirmed dry AMD, contain complement components, beta-amyloid, and other inflammatory factors [10]. Alternative pathway components and inhibitors as well as C3 have been shown to localize to the RPE, BrM, and choroid in humans [11]. This same tissue is also immunopositive for membrane attack complex (MAC) proteins, and it appears that MAC immunoreactivity is correlated with AMD severity and loss of RPE cells [12]. Furthermore, genotype-phenotype correlations have revealed associations between CFH risk alleles. Alleles that generate less effective CFH [13] result in peripheral drusen of increased size and reticular pigment [14], [15]. Conversely, the protective allele of CFB, which is a less efficient activator of the AP [16], is associated with smaller drusen and a reduced area covered by those drusen [17]. Taken together, there appears to be a correlation between the degree of pathology and amount of activation in the AP of complement.

The complement cascade is an integral part of the innate immune system, as it “complements” the ability of antibodies to clear pathogens or other “non-self” cells from an organism. There are three initiation pathways (classical, lectin, and alternative) involved, that all lead to a common terminal pathway [18]. The classical pathway (CP) is initiated when C1q, a pattern recognition molecule complex, binds to the surface of a pathogen or to an antibody:antigen complex. The lectin pathway (LP) can be initiated by the binding of a complex between mannan-binding lectin and mannan-binding lectin serine protease (MASP) or ficolin, and MASP to mannose-containing carbohydrates on bacteria, viruses, or unprotected cell surfaces. The alternative pathway (AP) is activated when either a spontaneously generated C3b molecule or one generated by either the CP or the LP binds to the surface of a pathogen or unprotected cell surfaces. All three pathways converge by forming a protease called C3 convertase, triggering the common terminal pathway. This final pathway is involved in executing the basic functions of the complement system. Convertase activity generates soluble anaphylatoxins involved in attracting macrophages and neutrophils, as well as cell-bound opsonins involved in removal of pathogens and cells. Finally, MAC formation, by virtue of its ability to form a non-specific pore in the cell membrane, is involved in cell lysis. Important for our study, the AP also provides an amplification loop for the CP and LP such that over 80% of C3 convertase deposited onto cells is derived from AP activation rather than the initiating pathway [19], [20]. Given the destructive capacity of the complement cascade, this system is tightly regulated by a number of inhibitors expressed by the host cells. These inhibitors include both cell-attached as well as soluble inhibitors that prevent complement activation on healthy self-cells. However, the levels as well as the cellular localization of these inhibitors are influenced by environmental factors, including oxidative stress [21] or cigarette smoke [22], [23].

Cigarette smoke is the only proven, modifiable risk factor for AMD. Previous studies have shown that smoking increases the risk of developing AMD 2–4-fold [24]. In addition, smoking also promotes the progression of AMD from the atrophic to neovascular form [25]. This progression may occur up to 10 years earlier in smokers compared to non-smokers [26]. However, cessation has been shown to reduce the risk of developing AMD and progression to the neovascular form [27]. The pathophysiology of cigarette smoke and AMD is complex and likely involves a number of different mechanisms. The RPE is under a high degree of oxidative stress from the turnover of photoreceptor outer segments. Cigarette smoke introduces many more free radicals [28] and likely contributes to oxidative damage loads. Smoking also depletes antioxidants (vitamin C, E, carotenoids [29]–[32], glutathione, cysteine, methylumbelliferone glucuronide, and ferroxidase [33], [34]) which act as natural oxidation inhibitors and help clear free radicals from the system. Cigarette smoke can directly activate C3, the primary constituent of the complement cascade, since it modifies C3 in a way that diminishes binding to CFH [35]. Lastly, nicotine found in cigarette smoke exerts a vasoconstrictive action via α-adrenergic receptor activation which reduces choroidal blood flow [36]. This reduction in choroidal circulation may underlie depositions in BrM due to inefficient clearance of debris from the RPE [37]. Taken together, although smoking increases the risk of AMD and leads to complement activation, there is no conclusive evidence that smoking-related pathology is the result of complement activation.

Given the aforementioned lines of evidence, we tested whether pathology is dependent upon complement activation by exposing mice to long-term cigarette smoke. Previous studies using the same model have documented activation of the terminal pathway of the complement system in the RPE and choroid, concomitant with damage to the RPE and photoreceptors [38]–[42]. However, little is known concerning which complement pathway might be involved. In the present study we investigated the role of the AP. We hypothesized that AP-deficient mice would be protected from smoke-induced deficits based on the observation that the majority of C3 activated on cell surfaces appears to be due to activity of the AP amplification loop (80–90% for CP [20] and LP [19]).

## Methods

### Animals

*CFB ^−/−^* mice on a *C57BL/6J* background were generously provided by V. Michael Holers (University of Colorado Health Science Center, Denver, CO). *C57BL/6J* (also referred to as wildtype [WT]) mice were purchased (Jackson laboratory, Bar Harbor, ME) [43]. Mice were confirmed to be negative for the RD8 locus by PCR using published primers (**Fig. S1**). See supplemental methods for details (**Text S1**). Animals were housed under a 12∶12 h, light:dark cycle with access to food and water *ad libitum*.

For electroretinography (ERG) or optical coherence tomography (OCT) imaging, mice were anesthetized using intraperitoneal injections of xylazine and ketamine (20 and 80 mg/kg, respectively), and pupils were dilated (2.5% phenylephrine HCl and 1% atropine sulfate). Hydroxypropyl methylcellulose (GenTeal, 0.3%) was used as an adherent for the contact lens electrode (ERG) or was applied regularly throughout the imaging process to maintain corneal hydration. All experiments were approved by the Medical University of South Carolina Institutional Animal Care and Use Committee and performed in accordance with the Association for Research in Vision and Ophthalmology statement for the use of animals in ophthalmic and vision research.

### Exposure to Cigarette Smoke

Eight-week-old *C57BL/6J* and *CFB ^−/−^* male mice were divided into two groups (n = 12 per group and genotype). The control group was kept in a filtered air environment, and the experimental groups were subjected to cigarette smoke. Cigarette smoke exposure (CE) was carried out (5 hours per day, 5 days per week) by burning 3R4F reference cigarettes (University of Kentucky, Louisville, Kentucky, USA) using a smoking machine (Model TE-10; Teague Enterprises) for 6 months. The average concentration of total suspended particulates was 130 mg/m^3^ and was monitored twice daily.

### Electroretinography

ERG recordings and data analyses were performed as previously described [44] using the EPIC-2000 system (LKC Technologies, Inc.). In short, mice were dark-adapted overnight prior to testing. ERG responses were obtained using light stimuli with varying light intensities and wavelengths. Under scotopic conditions, responses to 10 µs single-flashes of white light (maximum intensity of 2.48 cd m^−2^) between 40 and 0 db of attenuation were measured. After light-adapting animals for 2 min with rod-saturating light [45], UV-cone responses were tested using LED flashes centered at 360 nm. Peak a-wave amplitude was measured from baseline to the initial negative-going voltage, whereas peak b-wave amplitude was measured from the trough of the a-wave to the peak of the positive b-wave.

### Optokinetic Response Test

Visual acuity and contrast sensitivity of mice were measured by observing their optomotor responses to moving sine-wave gratings (OptoMotry) as previously described [46]. Mice reflexively respond to rotating vertical gratings by moving their head in the direction of grating rotation. To observe these movements, mice were placed individually on the central elevated pedestal surrounded by a square array of computer monitors that display stimulus gratings. Mice were monitored via an overhead closed-circuit TV camera that allowed the observer to view only the central platform and not the rotating grating. Mice were allowed to adjust to the chamber for 2 min with the monitors displaying a 50% gray uniform field prior to testing, and monitors returned to a homogenous gray between trials. All tests were conducted under photopic conditions with a mean luminance of 52 cd m^−2^. Visual acuity was measured by finding the spatial frequency threshold of each animal at a constant speed (12 deg/s) and contrast (100%) with a staircase procedure that systematically increased the spatial frequency of the grating until the animal no longer exhibited detectable responses. Contrast sensitivity was determined by taking the reciprocal of the contrast threshold at a fixed spatial frequency (0.131 cyc/deg) and speed (12 deg/s). It has previously been determined that this spatial frequency falls within the range of maximal contrast sensitivity for 9-month-old *C57BL/6J* mice (data not shown). Contrast of the pattern was decreased systematically in a staircase manner until the animal stopped responding.

### Quantitative RT-PCR

RPE/choroid/sclera (henceforth referred to as RPE/choroid) and retina fractions were isolated from control and smoke-exposed animals and stored at −80°C until they were used. Quantitative RT-PCR (QRT-PCR) analyses were performed as previously described in detail [47]. In short, real-time PCR analyses were performed in triplicate in a GeneAmp® 5700 Sequence Detection System (Applied Biosystems) using standard cycling conditions. Quantitative values were obtained by the cycle number, normalizing genes of interest to β-actin, and determining fold difference between room air and CE within genotypes. Fold difference values were compared using *Z*-test analyses, accepting a significance of *P*<0.05. See supplemental data for the full list of primers used (**Table S1**).

### SD-OCT Imaging

SD-OCT was performed using a Bioptigen Spectral Domain Ophthalmic Imaging System (Bioptigen Inc., Durham, NC). The system is equipped with a probe and platform designed for mice that allows for easy orientation and alignment of the central retina.

Imaging and data analysis were performed with Bioptigen® InVivoVue software. Rectangular volume scans were taken in the nasal quadrant from the optic disc, each volume consisting of 33 B-scans (1,000 A-scans per B-scan). Five separate scans were collected and averaged to generate a high resolution image. Vertical calipers were placed to measure the outer nuclear layer (ONL) and inner nuclear layer (INL) for each scan. All measurements were taken 500 µm from the optic disc. Data reflects the average of both eyes per animal.

### Tissue Preparation

The eyes were enucleated, and a slit was cut into the cornea to allow for rapid influx of fixative. Eyes were fixed overnight in 2.5% glutaraldehyde, 1% formaldehyde, 3% sucrose, and 1 mM MgSO_4_ in 0.1 M cacodylate buffer, pH 7.4. The eyes were then dissected and small central portions were osmicated for 60 minutes in 0.5% OsO_4_ in 0.1 M cacodylate buffer, processed in maleate buffer for *en bloc* staining with uranyl acetate, dehydrated in graded ethanols, and processed for resin embedding as in [48]. Serial sections were cut at 90 nm on a Leica Ultramicrotome onto carbon-coated Formvar® films supported by nickel slot-grids.

### Ultrastructural Analysis

Transmission electron micrographs (EMs) were captured using a JEOL JEM 1400 transmission electron microscope using SerialEM software to automate image capture overnight with 1200–1500 images captured per section, yielding datasets that were then processed with the NCR Toolset [49], [50] to generate image mosaics with corrections for image aberrations induced by electron microscopy.

Electron microscopy images were evaluated using Adobe® Photoshop® and ImageJ software. For each animal, two RPE cells were outlined using the apical processes and the basal lamina (thickness) as well as the basolateral walls (length) as borders. A masking-layer for all mitochondria present within an RPE cell was created to calculate average mitochondrial number and size. To determine mitochondrial position, the centroid coordinates for each mitochondrion was calculated as a percentage of the corresponding RPE length and thickness, respectively. Coordinates were assigned to basolateral, basal, central, or apical compartments based on normalized X-Y coordinates. The density of basal laminar (BL) infoldings was determined by dividing the area occupied by individual infoldings, by the total BL area for each of the two RPE cells. The BL area was isolated for each RPE cell in Photoshop®. Images were binarized, processed using a median filter (10 px radius) to reduce background noise, and analyzed for area measurements. BrM thickness was determined by analyzing ∼13 µm mean length of BrM adjacent to each of the outlined RPE cells. A masking layer was created over the inner collagenous layer (ICL) along each segment of BrM using RPE-BrM and the middle elastic layer (MEL) as boundaries. The area of the masking layer was divided by the length of the segment to determine an average BrM ICL thickness. For outer segment (OS) width, eight OS were randomly sampled from each animal, measuring the width at the thickest point and calculating an average. Müller cell percent area was determined by subtracting the area occupied by the rod/cone somas in a ∼600 µm^2^ area of the ONL and dividing by the total area analyzed; using the same masking technique as described from BrM thickness analysis.

Mitochondrial distribution profiles were generated by performing a percent normalization on the coordinates of centroids for all mitochondria with respect to the length/width of the RPE cell they were imaged from. After plotting the data, we assigned each mitochondrion to one of 4 bins as follows: basolateral (x-value <0.20 or x-value >0.80), basal (0.20≤ x-value ≤0.80 and y-value <0.33), central (0.20≤ x-value ≤0.80 and 0.33≤ y-value <0.66), and apical (0.20≤ x-value ≤0.80 and 0.66≤ y-value <1). Significance values were based on the percentage of mitochondria that fall into each bin.

### Immunohistochemistry

Paraffin-embedded mouse eyes were sectioned at 5 µm and immunostained for the presence of complement deposition using an antibody (Ab) to C3, split-fragment C3d (R&D Systems, Volcano, CA). Immunohistochemistry staining was performed as previously described [51]. In brief, primary-Ab binding was visualized using the Vector Laboratories ImmunoEdge detection kit and diaminobenzidinechromogen development (Vector Laboratories, Burlingame, CA). Specificity of staining was assessed by omission of primary Ab and the use of isotype controls. To assess the cellular and spatial localization of C3d immunostaining, eye sections were pretreated in 30% hydrogen peroxide for 18 hours to bleach melanin deposits prior to immunostaining, as previously described [52].

### Statistics

For data consisting of multiple groups, repeated measures analysis of variance (ANOVA) followed by the Fisher *post hoc* test (*P*<0.05) was used; single comparisons were analyzed using the Student *t*-test assuming equal variance (*P*<0.05; Statview; SAS Institute, Inc.). Fold changes in QRT-PCR experiments were analyzed using a *Z*-test (*P*<0.05).

## Results

### Effect of AP Deficiency on Smoke-induced Impairment of Visual Function

Dry AMD in patients is associated with thickening of BrM that can eventually lead to photoreceptor degeneration [53], [54]. Even without cell loss, the structural changes in BrM can lead to impaired nutrient exchange between the choroid and the RPE (reviewed in [55]), as well as a reduction in the generation and delivery of the vitamin-A-based chromophore, 11-*cis* retinal, to the photoreceptor outer segments [56]. In patients, these AMD-associated pathologies lead to a reduction in visual function as measured by ERG, as well as changes in visual acuity and contrast sensitivity.

**Figure 1:**
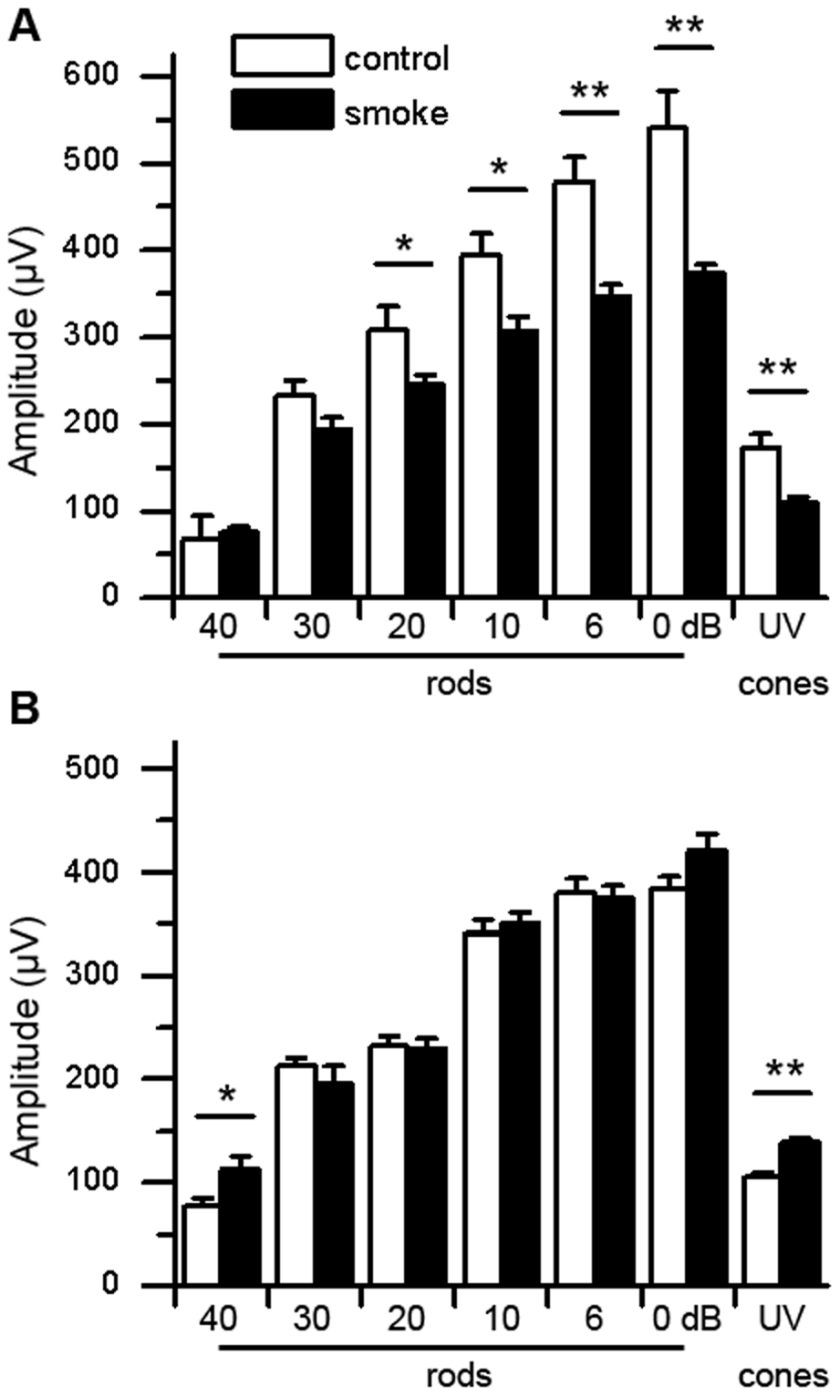
Electroretinography analysis for WT and *CFB* ^−/−^ mice following CE. ERG recordings were performed in cohorts of age-matched WT (**A**) and *CFB ^−/−^* (**B**) mice exposed to 6 months of cigarette smoke (CE) or room air. Dark-adapted, scotopic ERGs were recorded in response to increasing light intensities, and light-adapted photopic UV-cone ERGs to a single, maximum light intensity. Scotopic ERGs were analyzed using multiple ANOVA, followed by comparison of individual light intensities using *t*-test analyses. WT mice exposed to smoke had significantly lower dark-adapted b-wave amplitudes compared to controls (*P*<0.001), in particular at higher light intensities (20, 10, 6, 0 dB). Impairment in cone function is evidenced by the reduction in maximum UV-cone b-wave amplitudes. In comparison, ERG amplitudes in both scotopic and photopic ERGs were unaffected in AP-deficient mice exposed to smoke. Photoreceptor cell responses (a-waves), which drive the b-waves, were equally affected (data not shown). Data are expressed as mean ±SEM (n = 6–8 per condition; *, *P*<0.05; **, *P*<0.001).

Here, ERG recordings were assessed to determine the impact of 6 months of CE on mouse retinal function. ERG responses were analyzed under dark-adapted, scotopic conditions to determine rod photoreceptor function, as well as under photopic conditions after light-adaptation, to determine cone function. Here, the focus was on the mouse UV-cones, which have been shown to be more susceptible to perturbations (summarized in [57]) and can be isolated spectrally by electroretinography. For both scotopic and photopic ERGs, the b-wave amplitudes were reported, which are a mass potential generated by the bipolar cells that sum photoreceptor output (reviewed in [58]). Six months of CE caused an overall reduction in scotopic ERG amplitudes in WT animals (*P*<0.001) (**Fig. 1A**). While the b-wave responses of the smoke-exposed animals were marginally reduced when compared to non-exposed controls at lower light intensities (40, 30 dB), the differences reached significance at higher stimulus intensities (20, 10, 6, 0 dB; *P*<0.05). Likewise, the maximum UV-cone response after CE was reduced in WT mice by ∼30% (*P*<0.001). The photoreceptor cell response (a-wave), which drives the b-waves, was equally affected (data not shown). In contrast, photoreceptor cell function was significantly preserved in smoke-exposed *CFB ^−/−^* mice (*P* = 0.5), irrespective of whether rod or UV-cone function was assessed (**Fig. 1B**). However, while rod ERG amplitudes have previously been found not to differ between age-matched WT and *CFB ^−/−^* mice at 3-months-of-age [44], both rod and cone ERG amplitudes are reduced by 20–30% across all light intensities by 9-months-of-age (*P*<0.01). See supplemental data (**Fig. S2**).

**Figure 2:**
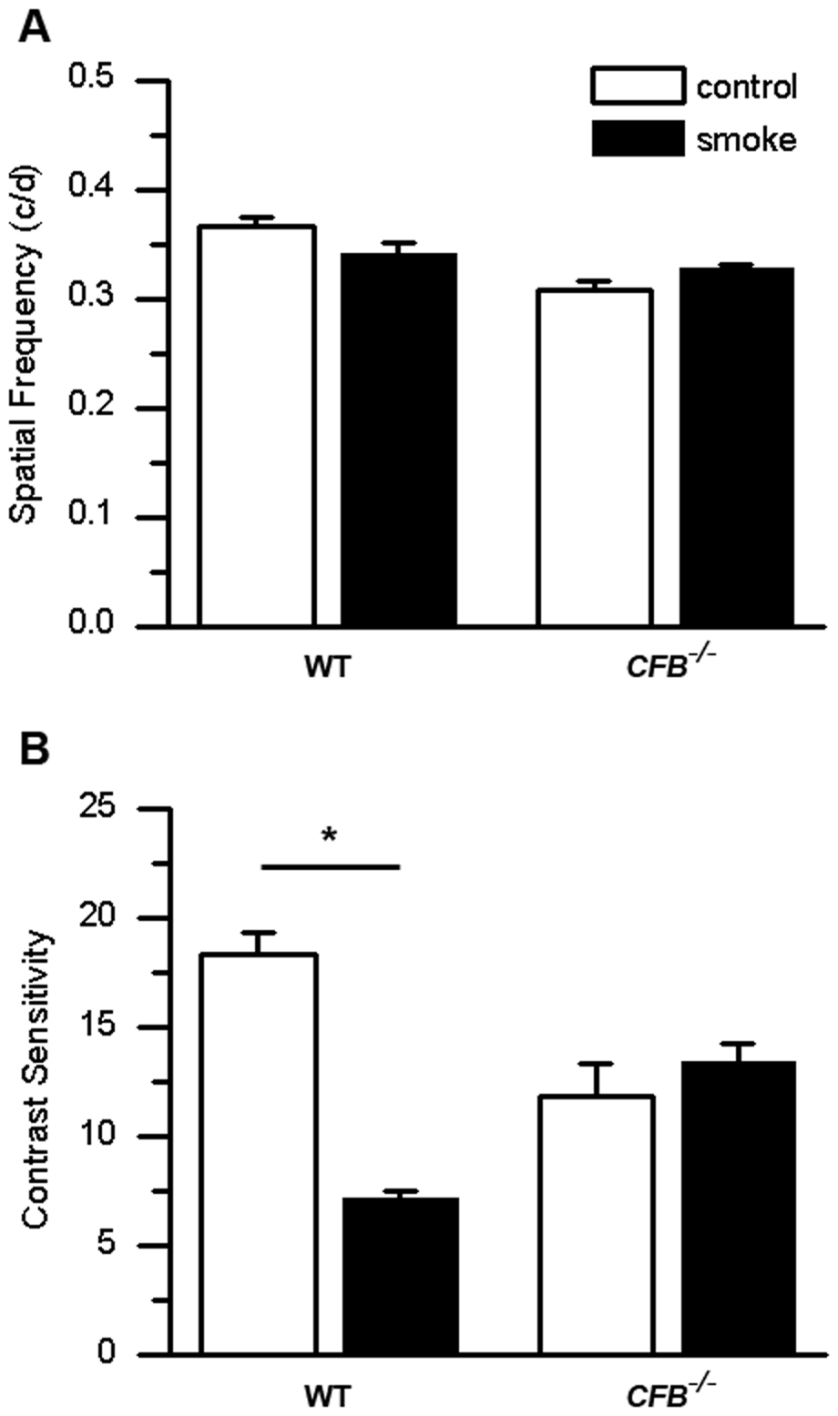
CE does not affect visual acuity, but impairs contrast sensitivity. Optomotor responses were analyzed in WT and CFB ^−/−^ mice after exposure to 6 months of cigarette smoke (CE) or room air. (A) Visual acuity was measured by identifying the spatial frequency threshold at a constant speed (12 deg/sec) and contrast (100%). Spatial frequency thresholds were not affected by treatment, although a genotype-dependent difference in visual acuity was identified (WT versus CFB ^−/−^ at room air, P<0.001). (B) Contrast sensitivity was measured by taking the reciprocal of the contrast threshold at a fixed spatial frequency (0.131 cyc/deg) and speed (12 deg/sec). We previously determined that this spatial frequency falls within the range of maximal contrast sensitivity for 9-month-old WT mice (data not shown). WT mice after CE showed a significant reduction in contrast sensitivity compared to controls, while AP-deficient mice remained unchanged. As for visual acuity, contrast sensitivity was affected by genotype (P<0.001), with CFB ^−/−^ mice being significantly less sensitive. Data are expressed as mean ±SEM (n = 3–9 per condition; *, P<0.01).

To correlate the ERG findings with a behavioral measure of visual acuity, we performed OKR tests to determine spatial frequency (**Fig. 2A**) and contrast sensitivity (**Fig. 2B**) under photopic light conditions. While spatial acuity and contrast sensitivity are influenced by both inner and outer retina function, both measures have been shown to correlate with photoreceptor cell function. Spatial acuity, which in rodents is low due to spatial summation, can be observed after RPE [59] or photoreceptor cell damage [60]. Likewise, loss of contrast sensitivity is a manifestation of photoreceptor cell loss in retinal degenerate mice (rd10) [61] or the Royal College of Surgeons rat [62]. Spatial frequency threshold (spatial acuity) in WT mice after CE did not differ from that of control animals exposed to room air (0.34±0.01 cyc/deg versus 0.37±0.01 cyc/deg; n.s.). Spatial frequency threshold was slightly lower in *CFB ^−/−^* control mice (0.31±0.01) than in WT controls (*P*<0.001); but just as in WT animals, *CFB ^−/−^* mice were unaffected by CE (0.33±0.00; P>0.05). Contrast sensitivity on the other hand was affected by smoke inhalation. A robust decrease in contrast sensitivity was observed for the WT smoke-exposed group (7.16±0.37) compared to controls (18.34±1.00; *P*<0.01). As for the remaining visual function tests, contrast sensitivity threshold was lower for *CFB ^−/−^* control mice (9.48±1.22) when compared to WT controls (*P*<0.001), but the levels were unaffected by CE (10.78±0.61; *P* = 0.34).

### Effect of AP Deficiency on Smoke-induced Changes in Gene Expression

**Figure 3:**
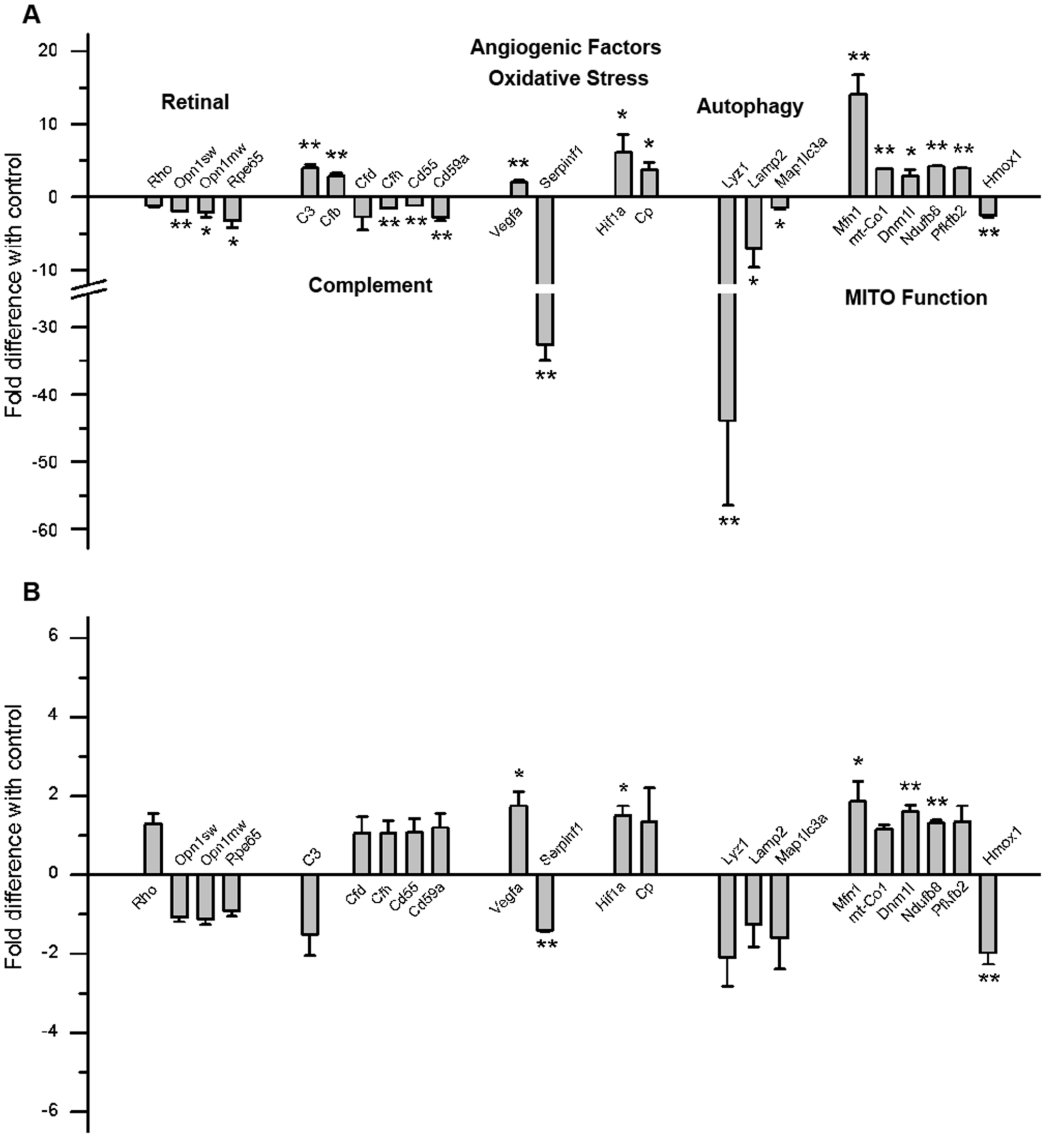
Gene expression changes in ocular tissues between WT and *CFB ^−/−^* mice following CE. Analysis of marker gene expression in WT (**A**) and *CFB ^−/−^* (**B**) mice, using quantitative RT-PCR on cDNA generated from RPE/choroid/sclera fraction and retina. Quantitative values were obtained by cycle number (*C*t value), determining the difference between the mean experimental and control (Actb) Δ*C*t values for cigarette smoke (CE) versus room-air-exposed mice within each genotype (fold difference). Candidates were examined from a number of categories including photoreceptor cell function (Rho, Opn1sw, Opn1mw, Rpe65), complement activation (C3, Cfb, Cfd, Cfh, Cd55, Cd59a), control of angiogenesis (Vegfa, Serpinf1), oxidative stress (Hif1a, Cp), autophagy (Lyz1, Lamp2, Klc3), and mitochondrial function (Mfn1, mt-Co1, Dnm1l, Ndufb8, Pfkfb1, Hmox1). Significant changes were identified in all six categories for WT mice, suggesting decreased cone function and chromosphere production, increased complement activation, the generation of a pro-angiogenic, and oxidative environment with impaired repair processes (autophagy) and reduced energy production under CE conditions. In comparison, gene expression was minimally affected in *CFB ^−/−^* animals. Data are expressed as mean ±SEM (n = 3 per condition; *, P<0.05).

To gain further insight into how CE might be affecting visual function, QRT-PCR was performed for a wide range of genes that fall into one of six categories: photoreceptor cell function, complement activation, control of angiogenesis, oxidative stress, autophagy, and mitochondrial function. With the exception of rod and cone opsin gene levels, which were determined in retina samples, all other genes reflected changes in the RPE/choroid fraction. The fold-difference between room-air and smoke-exposed animals are plotted for WT (**Fig. 3A**) and *CFB ^−/−^* mice (**Fig. 3B**).

In WT mice, CE significantly altered gene expression levels for genes belonging to all six categories. First, since CE reduced ERG-amplitudes and contrast sensitivity, levels of the genes encoding for the apoproteins of the photopigments as well as the rate-limiting enzyme for the production of the chromophore required for all pigments, RPE65, were examined. With the exception of rhodopsin (Rho), all photoreceptor function genes were significantly downregulated (short- and middle-wavelength cone opsins, Opn1sw; Opn1mw; Rpe65). Second, CE has been shown to increase complement activation in the mouse RPE [39]. Here, we find significant changes in gene expression in the RPE/choroid following CE for complement genes, including increases in essential components of the alternative and terminal complement pathway, Cfb and C3; and a decrease in complement inhibitors, Cfh, Cd55, and Cd59. Third, smoking is associated with edema, which in the RPE is controlled by two factors: the pro-angiogenic factor, VEGF (Vegfa), and the anti-angiogenic factor, PEDF (Serpinf1) [63]. Vegfa was found to be significantly elevated, whereas Serpinf1 showed a robust decrease of over 30-fold. Fourth, smoking produces carbon monoxide, which binds to hemoglobin, reducing oxygen availability, resulting in hypoxia. Here, we found that markers of oxidative stress, including the hypoxia-inducible transcription factor, Hif1a, and the ferroxidase enzyme, ceruloplasmin (Cp), were upregulated in the RPE/choroid of smoke-exposed mice. Fifth, autophagy is a basic catabolic process designed to remove damaged proteins and organelles expected to result from CE. Autophagy genes in the RPE were downregulated under smoking conditions, with significant differences found for lysozyme 1 (Lyz1), lysosomal-associated membrane protein 2 (Lamp2), and microtubule-associated protein 1 light chain 3 alpha (Map1lc3a). Sixth, an increase in mitochondrial number and decline in mitochondrial function has been reported in response to tobacco smoke [64]. In support of mitochondrial stress and biogenesis, we found a significant increase in mitochondrial genes in the RPE/choroid affecting mitochondrial fission and fusion (mitofusin 1, Mfn1; dynamin 1-like, Dnm1l); an increase in mitochondrial respiratory proteins (NADH dehydrogenase (ubiquinone) 1 beta subcomplex 8, Ndufb8, mitochondrially encoded cytochrome *c* oxidase I, mt-Co1), as well as a decrease in protective response gene heme oxygenase (decycling) 1 (Hmox1). Finally, 6-phosphofructo-2-kinase/fructose-2,6-biphosphatase 1 (Pfkfb1), the rate limiting enzyme in glycolysis, was found to be upregulated, potentially increasing ATP production via glycolysis.

In *CFB ^−/−^* mice, CE had minimal effects on retina and RPE/choroid gene expression. No differences were observed in mRNA levels of genes involved in photoreceptor cell function, complement activation or autophagy under CE conditions when compared to controls. Significant changes were observed in genes controlling angiogenesis (Vegfa, Serpinf1), oxidative stress (Hif1a) and mitochondrial function (Mfn1, Dnm1l, Ndufb8, and Hmox1); however, the fold changes were smaller than those in WT mice (5–84% of WT levels with a median of 31%). Since functional differences were observed by ERG and OKR between WT and *CFB ^−/−^* mice, gene expression was compared between genotypes without CE (**Fig. S3**). At 9 months-of-age, expression of opsin genes was increased, but levels of Rpe65 were significantly lower. In the absence of CFB, levels of Cfd were found to be significantly elevated, whereas angiogenesis and autophagy genes were not altered in a consistent manner. Finally, changes in mitochondrial gene expression were observed, with a reduction in Mfn1, Dnm1l, Ndufb8, and Hmox1 along with an increase in Hif1a.

**Figure 4:**
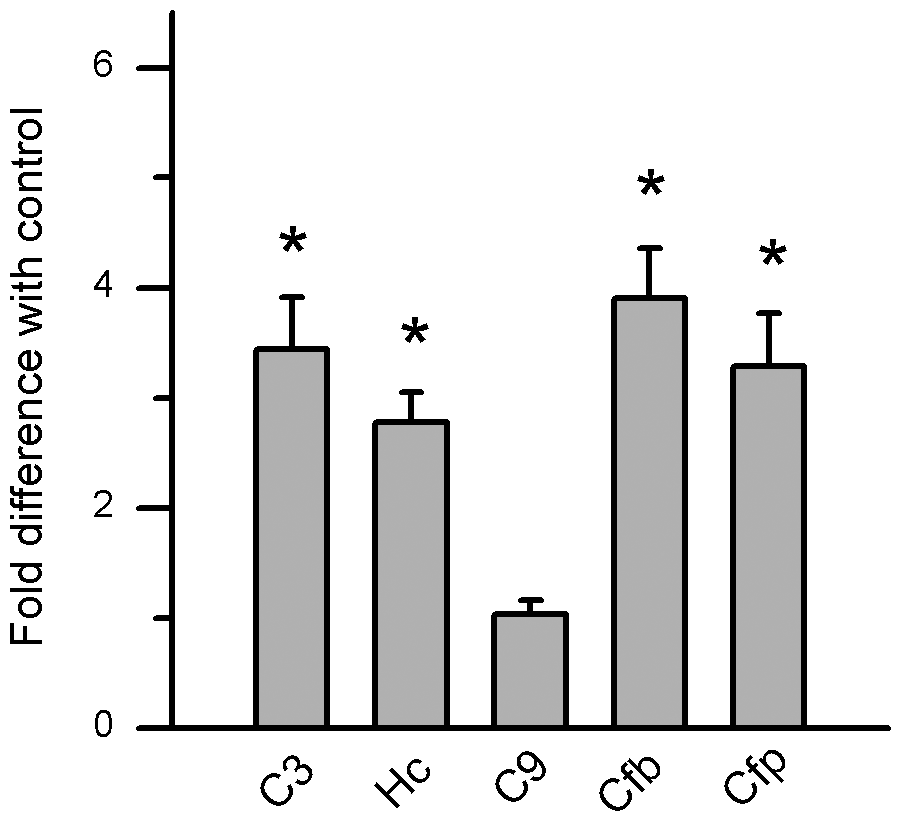
Gene expression changes in WT liver following CE. Analysis of complement gene expression in WT mice, using quantitative RT-PCR on cDNA generated from liver. Quantitative values were obtained by cycle number (*C*t value), determining the difference between the mean experimental and control (Actb) Δ*C*t values for cigarette smoke (CE) versus room-air-exposed WT mice (fold difference). Complement components C3 and C5 (hemolytic complement, Hc) were significantly elevated along with AP activators Cfb and Cfp, whereas C9 remained unchanged. Data are expressed as mean ±SEM (n = 3 per condition; *, P<0.001).

Since most complement components are generated in the liver, and many effects of complement activation are thought to be caused by systemic increases in complement activation, gene expression of C3, complement component 5 (C5), and complement component 9 (C9), as well as AP components Cfb and complement factor properdin (Cfp), were analyzed in livers from CE mice. With the exception of C9, gene expression of all other components was significantly elevated in WT mice (**Fig. 4**).

### Effect of AP Deficiency on Smoke-induced Histological Changes

**Figure 5:**
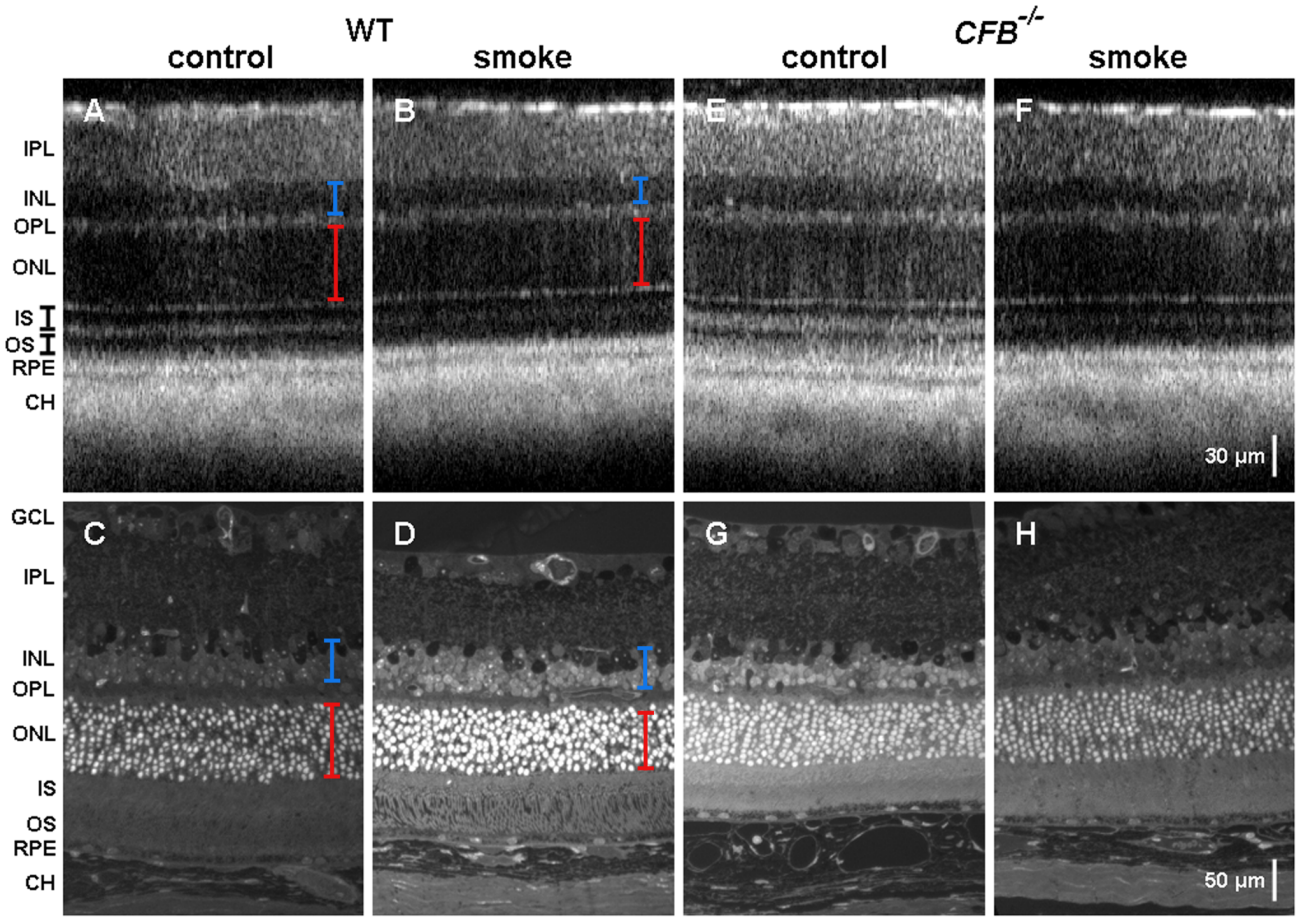
Optical coherence tomography and histological sections from WT and *CFB ^−/−^* mice following CE. Posterior poles from WT (**A**–**D**) and AP-deficient (**E**–**H**) mice were analyzed *in vivo* using OCT (**A,B** and **E,F**) and *ex vivo* using histology (**C,D** and **G,H**), comparing cigarette smoke (CE) and control conditions. OCT measurements were taken ∼0.5 mm from the optic nerve head in the nasal quadrant. There is visible thinning of the ONL (red) and INL (blue) in WT animals that is absent in *CFB ^−/−^* mice. Light microscopy performed on epoxy sections of central retina, derived from WT and *CFB ^−/−^* animals, supports the thinning observed in OCT images for WT mice exposed to smoke.

Given the functional and behavioral deficits described above, animals were examined using *in vivo* OCT imaging to determine the thickness of various retinal layers **Fig. 5AB, EF**. This was followed up with histological analyses by light microscopy **Fig. 5CD, GH** and EM (see the following sections). Representative OCT images are presented for WT and *CFB ^−/−^* mice under CE and room-air conditions, together with corresponding retinal tissue sections. There was noticeable thinning of the ONL and INL layer following CE in *C57BL/6J* animals that was readily observed in both OCT and epon sections. This change was not observed in *CFB ^−/−^* mice.

The OCT images were analyzed, using hyper-reflective bands to mark the beginning and end of each retinal layer of interest, as previously described [65]. We confirmed that there is a thinning of the ONL and INL in WT animals exposed to smoke when compared to age-matched controls (ONL: 53.04±0.57 versus 47.84±0.08; INL: 24.18±0.27 versus 21.41±0.72; *P*<0.03). In contrast, the slight reduction in ONL and INL thickness observed in *CFB ^−/−^* mice was not found to be statistically significant (ONL: 57.78±0.41 versus 51.95±0.64; INL: 24.29±0.49 versus 23.83±0.24; n.s.). ONL and INL thickness, however, did not differ between genotypes when animals were raised in room air.

### Ocular Localization of C3d after CE

**Figure 6:**
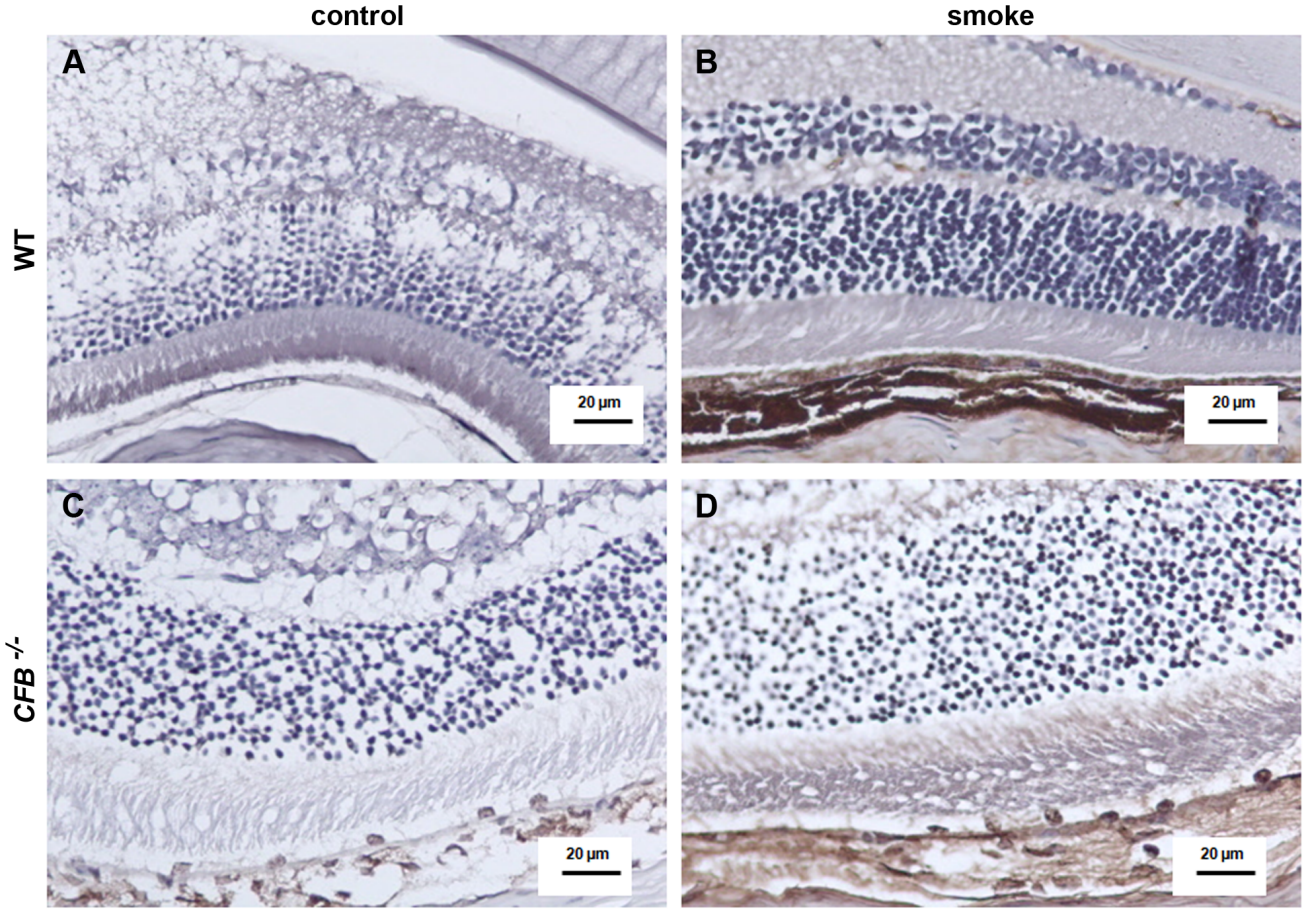
C3d deposition in eyes exposed to smoke. Localization of the complement activation product, C3d, one of the C3 opsonins that binds covalently to (cell) membranes was identified using immunohistochemistry, comparing WT (A,B) or *CFB ^−/−^* mice (C,D) exposed to room air (**A,C**) or cigarette smoke (CE) (**B,D**). Intense C3d immunoreactivity (brown deposits) was seen in RPE/BrM and choroid in smoke-exposed WT mice when compared to controls. In mice lacking the AP tick-over mechanism and amplification loop, reduced staining was observed in RPE/BrM and choroid after CE, whereas those animals exposed to room air demonstrated no immunoreactivity. Please note that in order to perform labeling in the pigmented RPE and choroid, melanin was bleached, resulting in faint pigmentation and revealing the nuclei of the RPE.

To correlate ocular pathologies with complement activation, we analyzed the localization of the complement activation product, C3d, a covalent cell membrane-attached breakdown product of C3 using immunohistochemistry. C3d was found to be deposited in RPE/BrM and choroid in WT mice exposed to smoke (**Fig. 6B**), but not in control animals (**Fig. 6A**). No immunoreactive material was found in the retina. Staining was attenuated in CFB ^−/−^ mice exposed to smoke (**Fig. 6D**), whereas the CFB ^−/−^ mice raised in room air were C3d negative (**Fig. 6C**).

### Lack of AP Abolishes Disruption of RPE-BrM Morphology Caused by Cigarette Smoke Exposure

**Figure 7:**
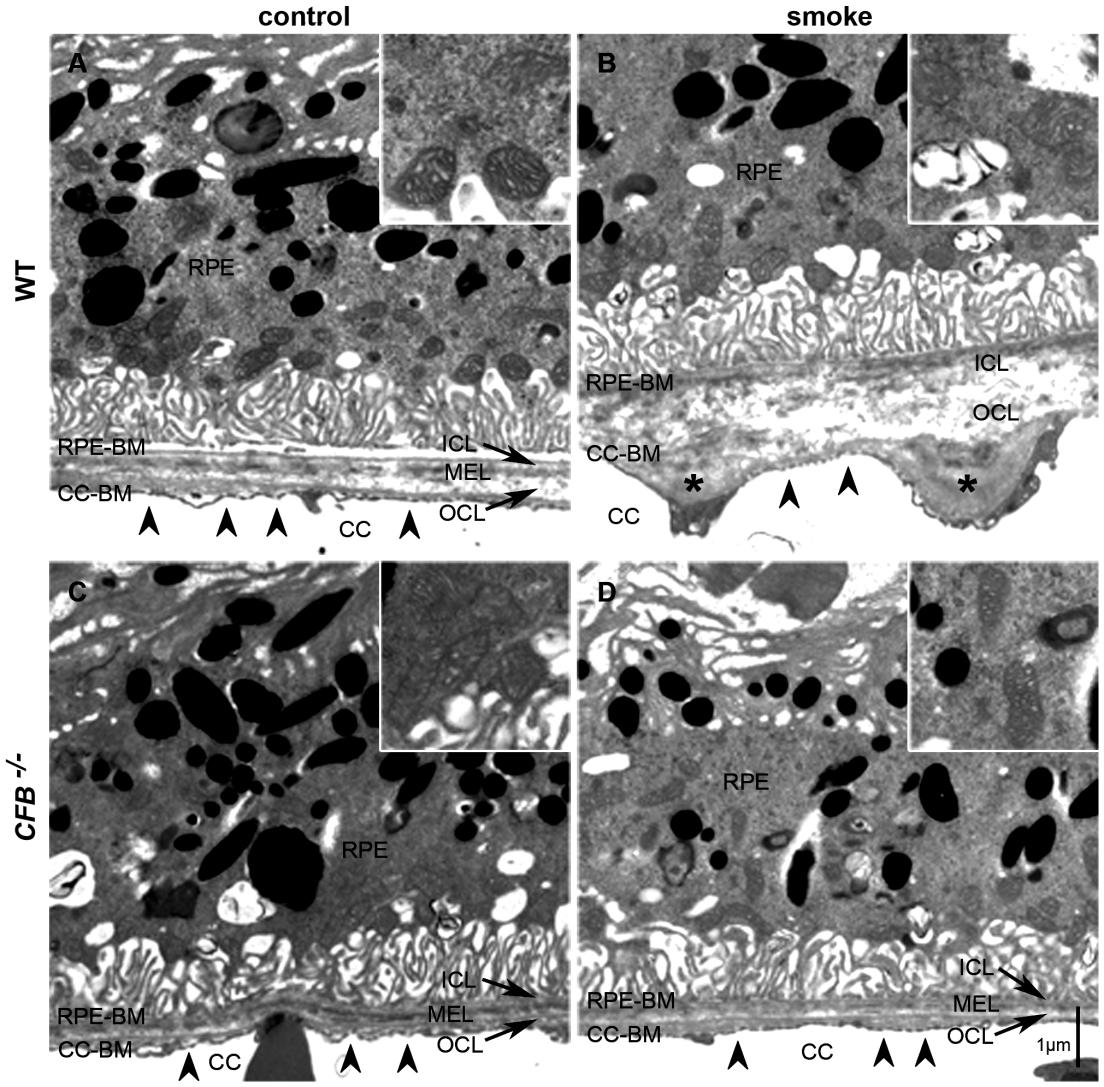
Ultrastructural changes in WT and *CFB ^−/−^* mice following CE. Electron micrographs of the RPE/BrM/choriocapillaris complex (RPE/BrM/CC) obtained from WT and *CFB ^−/−^* mice exposed to 6 months of cigarette smoke (CE) or room air were compared. (**A**) In a WT animal raised in room air, BrM exhibits an organized pentalaminar structure, consisting of RPE-BM, RPE basement membrane; ICL, inner collagenous layer; MEL, middle elastic layer; OCL, outer collagenous layer; CC-BM, choriocapillaris basement membrane; and the choriocapillaris endothelium has fenestrations along the entire membrane. (**B**) The RPE/BrM/CC in a WT animal exposed to smoke exhibits pathological changes. BrM is disorganized, losing its pentalaminar structure, and large deposits are present within the OCL. Note the presence of choriocapillaris fenestrations (*arrowheads*) overlying BrM of normal thickness, but fenestration loss and/or endothelial cell thickening adjacent to OCL deposits (*asterisks*). (**C**) The RPE/BrM/CC is not affected by the elimination of CFB, but is preserved in *CFB ^−/−^* mice exposed to smoke inhalation (**D**). *Insets* highlight the morphological features of mitochondria with degraded outer membranes and disorganized cristae in WT mice exposed to smoke and normal appearance in the other three samples.

In order to gain a better understanding of the ultrastructural deficits that underlie our AMD model, we analyzed EMs based on a number of different criteria. These include RPE cell morphology, BrM thickness, IS/OS length, and Müller cell area. Representative EMs for WT mice **Fig. 7AB** and *CFB ^−/−^* mice **Fig. 7CD** under control and CE conditions were depicted. BrM is a pentalaminar structure, consisting of the basement membrane of the RPE, the inner collagenous zone, the middle elastic layer (MEL), the outer collagenous zone, and the basement membrane of the choriocapillaris. Overall, BrM was remarkably thicker in smoke-exposed WT animals when compared to controls raised in room air. The typical pentalaminar structure of BrM was in disarray because the MEL can no longer be distinguished in the smoke condition. Also, note the large deposits in the outer collagenous layer (asterisks) in BrM that are absent in the controls. These deposits were associated with lower fenestration density (arrows) in the choriocapillaris. These fenestrations are responsible for shuttling nutrients and waste across BrM between the RPE and choroid. In comparison, fenestrations were dispersed evenly along the choriocapillaris in control animals. Finally, mitochondria (insets) appeared to display a damaged phenotype under smoke conditions. The outer membrane was not clearly defined and the cristae appeared disorganized. In comparison, *CFB ^−/−^* mice displayed none of the smoke-induced distortions in RPE morphology that were identified in the WT animals. BrM was found to remain intact, exhibiting the identifiable 5 layers, without any apparent thickening. There were no deposits found in BrM and fenestrations remained evenly spaced in the choriocapillaris. Lastly, mitochondria appeared unchanged, displaying clear outer membranes with organized cristae.

EM results were quantified for the criteria listed above (**Table 1**) focusing on aspects of energy metabolism in the RPE (mitochondria and BL-infoldings), nutrient and waste transport (thickness of BrM) and photoreceptor function (photoreceptor OS width and size of cell body). RPE cell morphology was drastically altered in WT mice exposed to smoke. Mitochondria were found to be significantly larger in *C57BL/6J* smoke-exposed animals compared to controls (*P*<0.04), and made up a larger percentage of the total RPE cell area (*P*<0.05). Both of these criteria remained unchanged after CE in *CFB ^−/−^* animals. The total number of mitochondria remained unchanged in both genotypes. Interestingly, we also observed a treatment-dependent percent volume reduction of the BL-infoldings under smoking conditions for both genotypes (*P*<0.02). A robust thickening of BrM was observed in WT mice exposed to smoke (*P*<0.05) that was absent in *CFB ^−/−^* mice. Finally, we determined the average width of the rod photoreceptor OS and the area in the ONL occupied by photoreceptor cell bodies. WT mice had significantly thinner rod OS following CE (*P*<0.05), and exhibited photoreceptor hypertrophy and/or Müller cell atrophy when compared to controls (*P*<0.02). In comparison, *CFB ^−/−^* photoreceptor OS width and cell body size was unaffected regardless of treatment.

**Table 1:** Quantification of morphological structures from WT and *CFB ^−/−^* mice following CE.

Criteria	WT Control	WT Smoke	CFB ^−/−^ Control	CFB ^−/−^ Smoke
MITO Number	31.66±2.17	32.50±2.44	31.25±3.13	33.05±1.67
MITO Area	29.94±1.95	40.21±3.75 †	33.88±2.08	33.86±1.05
BrM ICL Thickness	0.15±0.01	0.18±0.01 †	0.15±0.02	0.12±0.00
BL% Area	75.28±1.07	67.17±2.02 †	72.04±1.64	63.88±1.14 ††
OS Width	1.61±0.07	1.35±0.08 †	1.53±0.03	1.46±0.08
Müller Cell% Area	27.85±1.42	21.46±1.27 †	22.73±1.25	23.39±1.21

All area and length/width measurements are expressed in µm^2^ and µm, respectively. MITO, mitochondria; BrM, Bruch's membrane; ICL, inner collagenous layer; BL, basal laminar infoldings; OS, outer segment. Data are expressed as mean ±SEM (n = 4–6 per group; †, *P*<0.05; ††, *P*<0.01).

### Mitochondrial Relocalization Following CE is Absent in AP Knockout Animals

**Figure 8:**
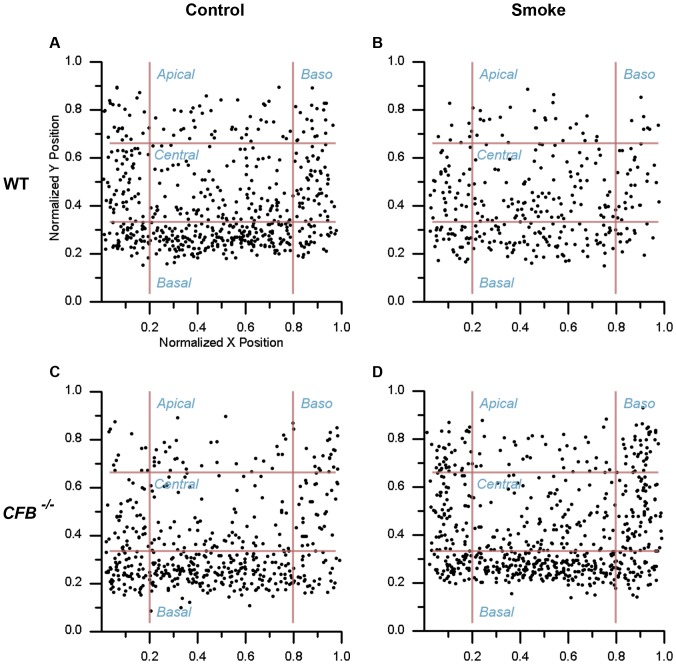
Mitochondrial localization is altered after CE. Mitochondrial position was determined from electron micrographs (depicted in Figure 7) by determining their centroid coordinates as a percentage of the corresponding RPE length and thickness, respectively. Each centroid was subsequently assigned to one of 4 bins (basolateral, basal, central, or apical). (**A**) The normalized positions of mitochondria within RPE of WT animals exposed to room air demonstrates that mitochondria are anchored predominantly along the basolateral and basal walls of the RPE cells and are more sparse throughout the central and apical portion (see text for more detail). (**B**) Cigarette smoke exposure (CE) affects the mitochondrial distribution in WT animals, with mitochondria exhibiting an apical shift from the basal to central compartment. (**C**) Mitochondrial distribution is not affected by genotype, with *CFB ^−/−^* mice raised under control conditions exhibiting a normal distribution profile. (**D**) Six months of CE had no effect on mitochondrial distribution in *CFB ^−/−^* animals.

Organelles in the RPE are distributed throughout the cell in a unique fashion: that is, mitochondria are localized along the basal and basolateral membranes, whereas melanocytes are localized predominantly along the apical surface. Mislocalization of organelles has been recognized as a hallmark of damaged RPE cells [66]. After 6 months of CE, melanocyte distribution was found to be unaltered in both genotypes (data not shown). The mitochondrial distribution profile for WT control mice confirmed that mitochondria are localized along the basal (33.2±2.7%) and basolateral (40.9±2.7%) sectors of RPE cells (**Fig. 8A**), whereas the central and apical sectors contain only 19.1±1.8% and 6.7±1.0% of the mitochondrial population, respectively. Following 6 months of CE, we noticed an apical shift of the mitochondria from the basal sector to the central sector (26.3±4.2%, 27.5±4.2%, respectively; *P*<0.05) (**Fig. 8B**). The basolateral and apical zones did not differ from the controls. The distribution profile for *CFB ^−/−^* room-air controls exhibited a similar mitochondrial distribution pattern as the *C57BL/6J* mice, with the majority of mitochondria sequestered in the basal (38.9±1.4%) and basolateral (40.6±2.8%) sectors (**Fig. 8C**). The central and apical zones were also similar, containing 14.0±2.3% and 6.4±0.9% of the mitochondrial population, respectively. The apical shift observed in *C57BL/6J* animals was absent in the *CFB ^−/−^* mice (**Fig. 8D**) following 6 months of smoke exposure. The percent distribution in smoke-exposed animals between basal, central, apical, and basolateral zones did not differ from age-matched room-air raised controls.

## Discussion

The main results of the current study were as follows: (1) WT mice exposed to long-term smoke inhalation exhibited functional (ERG) and behavioral (OKR) deficits in retinal function; (2) alterations in gene regulation in pathways involved in photoreceptor signal transduction, as well as a RPE cell metabolism, complement activation, angiogenesis, and catabolism may underlie these observed differences; (3) CE caused morphological changes in RPE-BrM that are similar to those observed in AMD such as thickening of BrM, deposit formation in BrM, loss of BL-infolding of the RPE and dislocation of mitochondria; (4) these changes in RPE-BrM may result in impairment of photoreceptor cell integrity as evidenced by retinal thickness changes in OCT and photoreceptor morphometric analyses in electron microscopy; (5) *CFB ^−/−^* animals appear to be protected from almost all smoke-induced changes that were observed in WT animals.

Dry AMD is considered a progressive disease, resulting in the gradual loss of vision. The earliest clinical hallmarks observable are either the appearance of drusen above a critical size (≥63 µm) and/or pigmentary changes in the center of the globe [1]. Drusen are deposits made up of proteinaceous and lipid debris, and are located between the RPE and the inner collagenous layer of BrM. Since alterations due to dry AMD and the normal aging processes overlap, it is difficult to assign observed alterations to one or the other. Additional changes that occur include the progressive thickening of both the inner and outer collagenous layer of BrM, including the development of inclusions in the collagenous and elastin layers of BrM [67]. In areas of RPE damage, choriocapillaris alterations, including reduction in fenestration or dropout, have been observed [68]. These alterations in the choriocapillaris may be the underlying reason for the impaired perfusion recognized in AMD [69]. Thickening of BrM, seen when using fluorescein angiography, has been postulated to act as a diffusion barrier between the choroid and BrM in AMD patients [70], which is supported by the observation of age- and lipid-related changes in permeability of BrM for macromolecules [71], [72]. Loss of photoreceptor function and structure is associated with dry AMD. In particular, abnormalities in dark adaptation of both rods and cones [73], with raised cone thresholds in the parafoveal region [74], have been observed. Loss of function may be due to retinoid deficiency based on the changes in BrM permeability. This hypothesis is supported by experiments conducted by Owsley *et al.* [56], demonstrating rod-mediated dark adaptation can be improved by high doses of oral retinol. However, dropout of photoreceptors has been reported in dry AMD starting in the parafoveal region, affecting both rods and cones [75].

The only environmental agent unequivocally linked to AMD is cigarette smoke [76], and epidemiological studies have linked smoking to the progression of AMD [77]. The most likely target for toxicity is the RPE, presumably by generating oxidative stress. Although the RPE is constantly exposed to toxic oxygen intermediates, it has available effective defenses against oxidative damage, including high amounts of anti-oxidants [78]. The RPE anti-oxidative capability is reduced with age [79], [80], and exposure to cigarette smoke may accelerate this normal age-related decline in RPE cell function.

Dry AMD has long been associated with inflammation and the complement cascade [12]. However, not until the association of single nucleotide polymorphisms (SNPs) in the AP inhibitor, CFH, with AMD risk, did that idea gain traction [4]–[7]. While there are other SNPs in genes that belong to the complement cascade (reviewed in Hecker and Edwards [81]), the strongest link is with CFH. Additional support linking CFH to AMD comes from patients with membranoproliferative glomerulonephritis (MPGN) type II, a disease associated with CFH mutations, who develop drusen which are indistinguishable from those in AMD [82]. Smoke extract has been shown to be able to activate the AP *in vitro* [35] and serum levels of complement components are elevated in smokers [83]. In a previous mouse study, using a comparable smoke exposure model, components of the complement pathway were identified between RPE and choroid in smoke-exposed, but not control animals [39]. Those components included the anaphylatoxin C3a, complement component C5, the MAC, as well as CFH. Thus, it appears likely that an increased activity in the AP, both systemically and locally, may mediate the AMD-like changes observed in the RPE/choroid and retina in animals exposed to constant smoke.

Here, we examined the effects of long-term CE on retinal structure and function in mice. Similar to AMD patients, we noticed a decrease in b-wave amplitudes under both scotopic and photopic conditions in WT mice exposed to smoke. In particular, under dark-adapted conditions, ERG amplitudes were diminished at light intensities sufficient to stimulate both rods and cones (mesopic range). In addition, contrast sensitivity under photopic conditions was reduced, while spatial acuity at maximum contrast was unaffected. The results of the QRT-PCR analysis allowed us to investigate several possible hypotheses. One scenario is that smoke exposure may reduce the amount of pigment available to trigger the phototransduction cascade. Gene expression levels for rhodopsin were found to be unaltered by smoke exposure whereas mRNA levels for UV- and MWL-cone opsin were both reduced approximately 2-fold; correlating with the functional results. A second potential reason for the reduction in function is that smoking reduces the amount of the rate-limiting enzyme, RPE65, required to generate the chromophore, 11-*cis* retinal. The QRT-PCR data revealed an approximately 3-fold decrease in Rpe65 mRNA levels following CE, which if translated into a 3-fold reduction in protein level would significantly impact chromophore formation [84]. Taken together, these results suggest that both cone apoprotein and chromophore production is reduced in smoke-exposed animals, leading to the observed decline in visual function in mesopic and photopic ranges. Future experiments are required to determine whether a reduction in cone opsin gene expression translates into shorter cone OS, or a drop-out in cones. However, the decrease in visual function following CE was accompanied by a thinning of both the ONL and INL as determined by OCT. Interestingly, in clinical studies, the thickness of the ONL has been shown to highly correlate with visual acuity in patients diagnosed with dry AMD [85]. Here, we cannot conclude that the thinning is due to cell loss; cell counts would need to be completed to further support this hypothesis. An alternative interpretation in light of the EM data, showing an apparent Müller cell atrophy (Table 1), might be that the cell bodies of the photoreceptors are equal in number, but closer together, resulting in a thinner ONL.

A growing body of literature suggests that dysfunctional mitochondria may lie at the core of AMD etiology [86], [87]. Feher *et al.* [88] have reported age-related changes in mitochondrial morphology that are accentuated in AMD. Aged RPE contain mitochondria that show membrane disorganization, a focal loss of cristae, and disruption of their apical-basal alignment. Work by He and Tombran-Tink [89] showed that cultured RPE cells from aged donor eyes contain large, fused mitochondria that generate less ATP than those obtained from young donors. Interestingly, exposing mice to smoke replicates many of the changes seen in the mitochondria of aged RPE cells. We found that CE leads to mitochondria that display a damaged phenotype, with fractured outer membranes and disorganized cristae. In addition, mitochondria were found to be significantly enlarged (**Table 1**). In support of the balance between mitochondrial fission and fusion being tipped towards fusion, Mfn1 was found to be increased ∼14-fold, whereas the fission protein, Dnm1l, was only increased 3-fold in the RPE of smoke-exposed animals. Since larger mitochondria need more mitochondrial respiratory proteins, it was logical to find that Ndufb8 and mt-Co1 were both upregulated 4-fold. Although mitochondrial respiration has not yet been measured in RPE cells obtained from smoke-exposed mice, mitochondrial ATP production is expected to be lower. In support of this hypothesis, we observed that gene expression for Pfkfb1, the rate-limiting enzyme in glycolysis, was found to be significantly increased. An increase in ATP production via glycolysis, an anaerobic redox reaction, is often observed under conditions of limited ATP synthesis by oxidative phosphorylation such as oxidative stress (Warburg effect [90]). Mitochondria are mobile organelles that tend to sequester in areas of high metabolic demand [91]. However, they can also move in response to physiological changes, which we observed in the mitochondrial distribution analysis. Taken together, we hypothesize that an inability to power the cellular machinery used to generate chromophore and shuttle nutrients and waste between the RPE and choroid, a consequence of defective mitochondria, might contribute to the visual defects that develop in the smoke-exposed animals.

In addition to these mitochondrial deficiencies in the RPE, we observed a thickening of BrM and formation of outer collagenous deposits. These deposits were similar to the ones found by Mettu *et al.*, using long-term exposure of mice to hydroquinone, an abundant oxidant in cigarette smoke, but differ in location from deposits in dry AMD [55]. In addition, alterations in the choriocapillaris such as a loss of fenestrations in areas opposing the large BrM deposits were observed. These morphological alterations may impede the aforementioned transport of nutrients and waste between the RPE and choroid, ultimately leading to impairment of photoreceptor cell function and structure. It is unclear as to the source of the material accumulating in BrM. However, as all markers of autophagy, the catabolic process responsible for removing damaged proteins and organelles, were all significantly down-regulated in the RPE from smoke-exposed animals. The increased amount of undigested material may get exocytosed on the basal side of the RPE, resulting in deposit formation. Interestingly, Wang *et al.* [39] reported a decrease in lysosomal activity in a culture model of aged RPE cells, and suggested that “the release of intracellular proteins via exosomes by the aged RPE may contribute to the formation of drusen.”

As indicated above, smoke exposure has been shown to be associated with an increase in complement activation in human patients [83], an increase in the risk for AMD [76], and an increase in complement deposition in the mouse eye [39]. Here, we found complement C3d deposition on the basal side of the RPE, indicating complement activation. Furthermore, we demonstrated increased complement gene expression for C3 and Cfb, a significant decrease in the membrane-bound and fluid phase inhibitors (Cd55, Cd59, and Cfh) in the RPE, as well as increased expression of AP complement proteins, C3 and C5 in the liver. Most importantly, we found that the great majority of the functional and structural alterations triggered by CE were absent in mice that lack the AP of complement activation. These mice have a functional CP and LP, but lack the tick-over process of spontaneous AP activation, as well as the AP amplification loop. Our observations in *CFB ^−/−^* mice can be paralleled by data from patients with the CFB protective (R32Q) and CFH risk (Y402H) alleles. The R32Q allele, which results in a CFB with less activity [16], is correlated with reduced drusen size and total drusen area [17]. Likewise, the Y402H allele, which binds less effectively to malondialdehyde epitopes and oxidized phospholipids (generated by oxidative stress [92], [93]), is correlated with the size of peripheral drusen and increases in reticular pigment [14], [15]. While both the human and our mouse data presented here suggest that increased AP activation is mediating structural damage in RPE/BrM, a contribution of altered gene expression and hence altered cellular homeostasis generated by R32Q, Y402H, or the lack of CFB (**Fig. S3**) cannot be excluded. Complement effector systems are capable of inducing inflammation, driving direct cell injury, and bridging the innate and adaptive immune system through the interaction of complement opsonins and anaphylotoxins. The precise mechanisms of complement-mediated injury herein is difficult to determine, and further studies need to be conducted to investigate the contributions of upstream and downstream components of the complement cascade on smoke-induced pathologies. To date, the presence of C3 activation fragments and MAC deposition in CE mice, together with the noted amelioration of disease in CE *CFB ^−/−^* mice supports the hypothesis that AP-mediated activation of the complement system plays a role in CE-induced AMD pathology, through complement-mediated effector mechanisms. However, a clear and sole contribution of the MAC cannot be ascertained, given that complement opsonins and anaphylatoxins can promote inflammation, cytokine release, and bridge adaptive immune responses that may progress AMD pathology independent of direct complement-mediated lysis. Finally, our group has generated a novel recombinant form of the AP inhibitory protein, CR2-fH, consisting of the AP-inhibitory domain of mouse CFH, linked to a complement receptor 2 (CR2) targeting fragment that binds complement activation products. CR2-fH has been used successfully to inhibit mouse choroidal neovascularization, a process requiring AP activation [44]. It would be of great interest to test this novel therapeutic to determine if smoke-induced retinal damage is preventable and/or reversible.

Taken together, there is growing evidence linking oxidative stress and smoking, as well as complement activation to the development and progression of AMD. Our data provided here show that CE in mice leads to functional and morphological defects in the retina, RPE, BrM, and choriocapillaris, also seen in patients with dry AMD. Our experiments, utilizing mice that lack the AP of complement activation, provide the first direct evidence that complement activation is required for these functional and structural alterations to occur, and suggest that these morphological alterations may be amenable to anti-complement-based therapies.

## Supporting Information

Figure S1 **Genotyping for CFB and RD8 in WT and CFB ^−/−^ mice.** DNA was isolated from WT and *CFB ^−/−^* mice, amplified with PCR primers, and resolved on a 1.5% agarose gel containing ethidium bromide for one hour. Gels were visualized under UV light. (A) PCR primers for complement factor B (CFB) were used to confirm the presence or absence of CFB. Lane 1 represents a GeneRuler 100 bp Plus DNA ladder. Amplified DNA samples from WT mice reveal a band at 748 bp which corresponds to the Amplicon CFB wild type allele (lane 2), whereas *CFB ^−/−^* mice reveal a band at 610 bp which corresponds to the CFB knockout allele (lane 3). (**B**) PCR primers for RD8 were used to confirm that both genotypes were RD8 positive. Lane 1 represents a 50 bp HyperLadderV. Amplified DNA samples from WT mice reveal a band at 220 bp which corresponds to the Amplicon RD8 wild type allele (lane 2) and no RD8 mutant band (lane 3). Amplified DNA samples from *CFB ^−/−^* mice show the same genetic profile (lane 4, 5).
(TIF)

Figure S2 **Electroretinography analysis for WT and CFB ^−/−^ mice at baseline.** ERG recordings were performed in cohorts of age-matched WT and CFB ^−/−^ mice exposed to room air for 6 months. Dark-adapted, scotopic ERGs were recorded in response to increasing light intensities, and light-adapted photopic UV-cone ERGs to a single, maximum light intensity. Using multiple ANOVA, CFB ^−/−^ mice had lower dark-adapted b-wave amplitudes compared to WT mice (P<0.05), although when individual light intensities were compared by t-test analysis, significance was only obtained at the highest light intensity (0 dB), suggesting that rod function is largely intact. However, cone function does appear to be impaired in CFB ^−/−^ mice as evidenced by the reduction in maximum UV-cone b-wave amplitude. Photoreceptor cell responses (a-waves), which drive the b-waves, were equally affected (data not shown). Data are expressed as mean ±SEM (n = 6–8 per condition; *, P<0.001).
(TIF)

Figure S3 **Differences in ocular gene expression between WT and** ***CFB ^−/−^*** **mice at baseline.** Analysis of marker gene expression in WT and *CFB ^−/−^* mice, using quantitative RT-PCR on cDNA generated from RPE/choroid/sclera fraction and retina. Quantitative values were obtained by cycle number (*C*t value), determining the difference between the mean experimental and control (Actb) Δ*C*t values for *CFB ^−/−^* versus WT mice exposed to room air (fold difference). Candidates were examined from a number of categories including photoreceptor cell function (Rho, Opn1sw, Opn1mw, Rpe65), complement activation (C3, Cfb, Cfd, Cfh, Cd55, Cd59a), control of angiogenesis (Vegfa, Serpinf1), oxidative stress (Hif1a, Cp), autophagy (Lyz1, Lamp2, Klc3), and mitochondrial function (Mfn1, mt-Co1, Dnm1l, Ndufb8, Pfkfb1, Hmox1). Significant differences were identified in all six categories for *CFB ^−/−^* mice, suggesting increased cone function and reduced energy production compared to WT animals. Data are expressed as mean ±SEM (n = 3 per condition; *, P<0.005; **, P<0.001).
(TIF)

Text S1 **Detection of CFB and RD8 Genotypes by PCR.**
(DOCX)

Table S1 **Quantitative RT-PCR primer sequences.**
(XLSX)
