# Supplementary figures and images for: Alternative Complement Pathway Deficiency Ameliorates Chronic Smoke-Induced Functional and Morphological Ocular Injury
Source: PLoS One. 2013 Jun 25;8(6):e67894. doi: 10.1371/journal.pone.0067894 (PMC3692454; doi:10.1371/journal.pone.0067894)

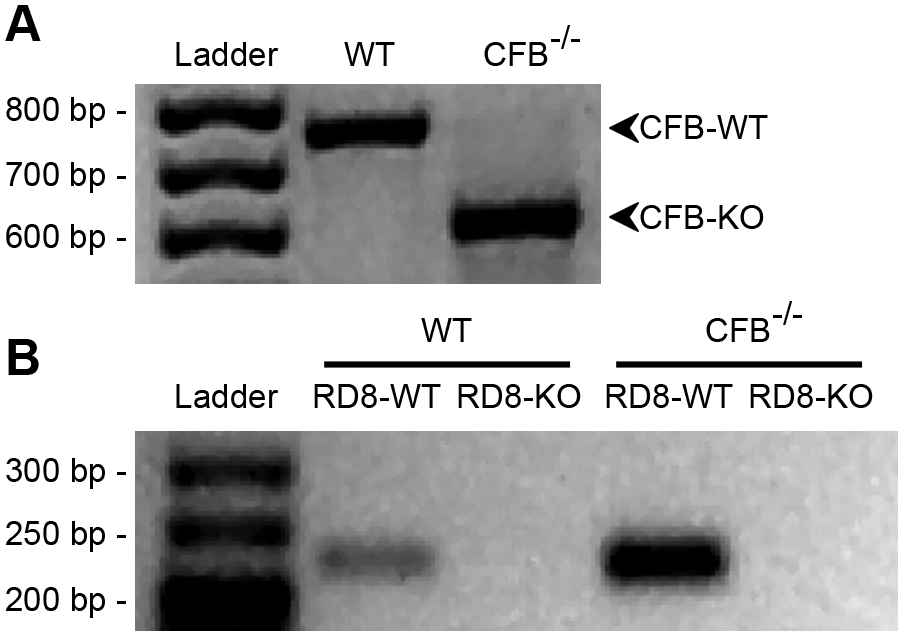

Supplement: Figure S1 — Genotyping for CFB and RD8 in WT and CFB −/− mice. DNA was isolated from WT and CFB −/− mice, amplified with PCR primers, and resolved on a 1.5% agarose gel containing ethidium bromide for one hour. Gels were visualized under UV light. (A) PCR primers for complement factor B (CFB) were used to confirm the presence or absence of CFB. Lane 1 represents a GeneRuler 100 bp Plus DNA ladder. Amplified DNA samples from WT mice reveal a band at 748 bp which corresponds to the Amplicon CFB wild type allele (lane 2), whereas CFB −/− mice reveal a band at 610 bp which corresponds to the CFB knockout allele (lane 3). (B) PCR primers for RD8 were used to confirm that both genotypes were RD8 positive. Lane 1 represents a 50 bp HyperLadderV. Amplified DNA samples from WT mice reveal a band at 220 bp which corresponds to the Amplicon RD8 wild type allele (lane 2) and no RD8 mutant band (lane 3). Amplified DNA samples from CFB −/− mice show the same genetic profile (lane 4, 5). (TIF) [file pone.0067894.s001.tif]

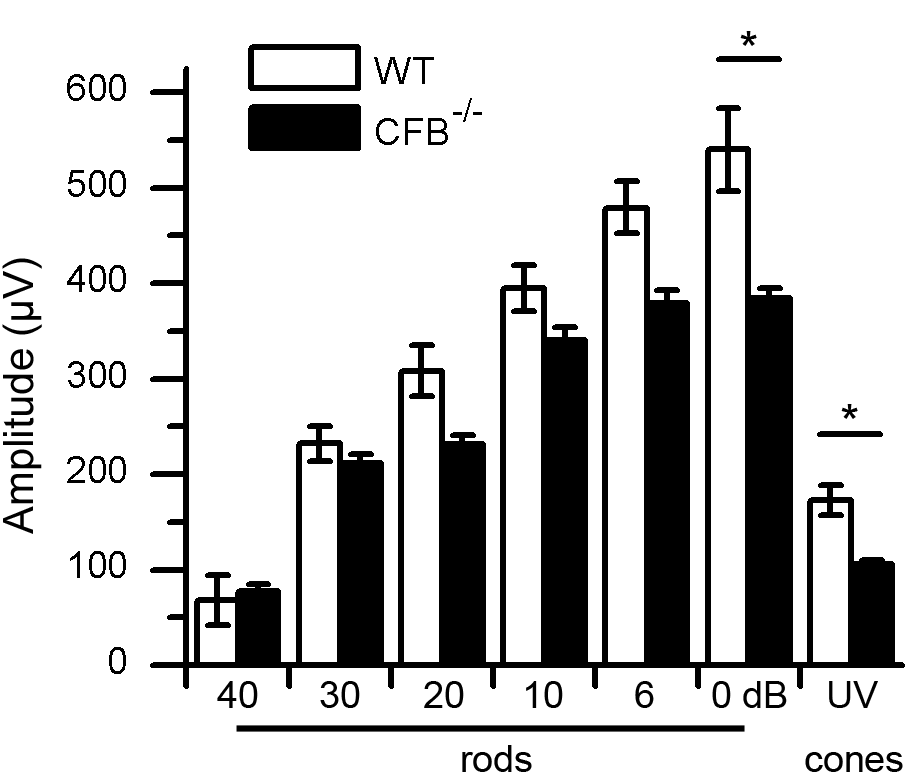

Supplement: Figure S2 — Electroretinography analysis for WT and CFB −/− mice at baseline. ERG recordings were performed in cohorts of age-matched WT and CFB −/− mice exposed to room air for 6 months. Dark-adapted, scotopic ERGs were recorded in response to increasing light intensities, and light-adapted photopic UV-cone ERGs to a single, maximum light intensity. Using multiple ANOVA, CFB −/− mice had lower dark-adapted b-wave amplitudes compared to WT mice (P<0.05), although when individual light intensities were compared by t-test analysis, significance was only obtained at the highest light intensity (0 dB), suggesting that rod function is largely intact. However, cone function does appear to be impaired in CFB −/− mice as evidenced by the reduction in maximum UV-cone b-wave amplitude. Photoreceptor cell responses (a-waves), which drive the b-waves, were equally affected (data not shown). Data are expressed as mean ±SEM (n = 6–8 per condition; *, P<0.001). (TIF) [file pone.0067894.s002.tif]

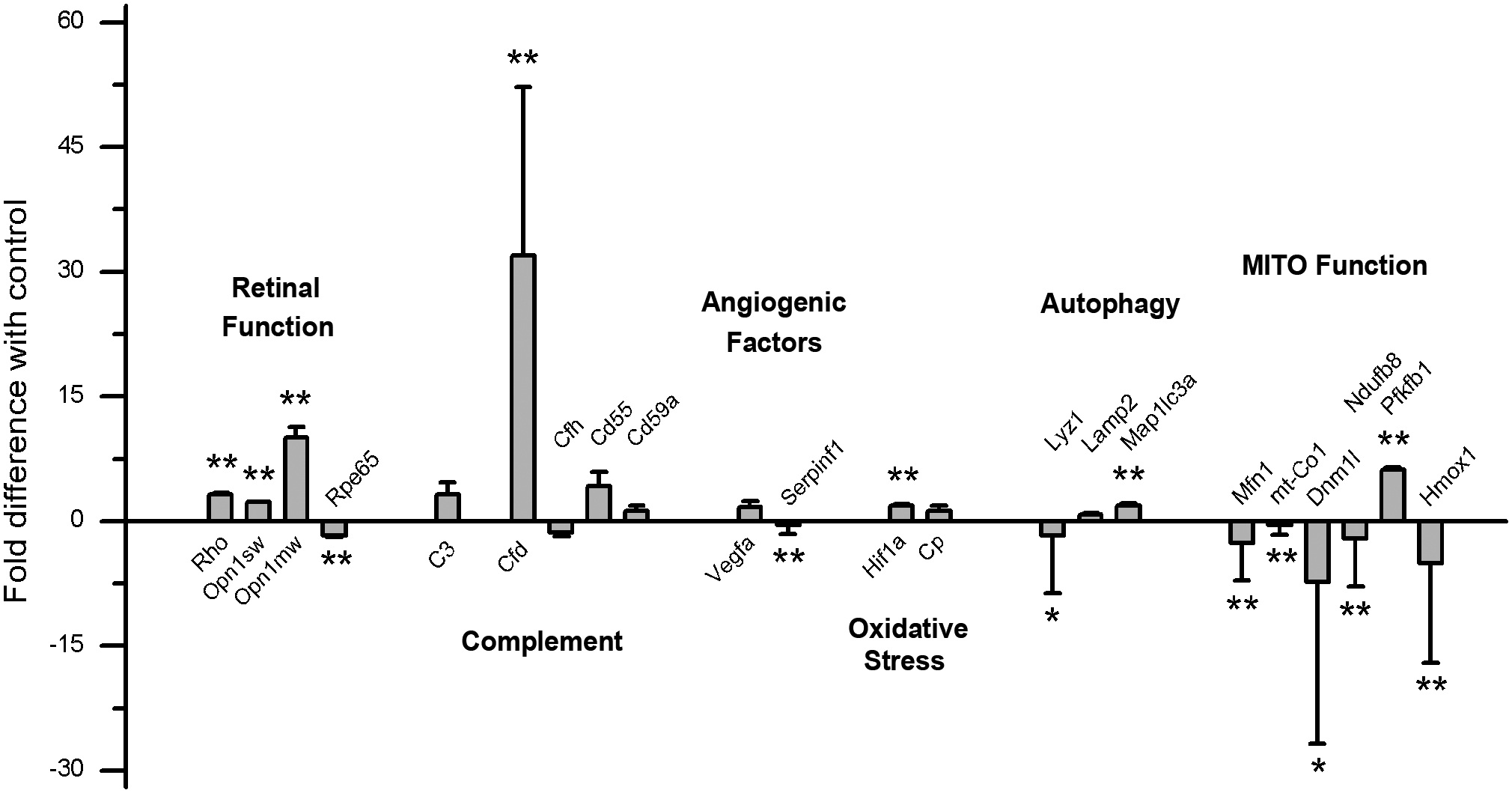

Supplement: Figure S3 — Differences in ocular gene expression between WT and CFB −/− mice at baseline. Analysis of marker gene expression in WT and CFB −/− mice, using quantitative RT-PCR on cDNA generated from RPE/choroid/sclera fraction and retina. Quantitative values were obtained by cycle number (Ct value), determining the difference between the mean experimental and control (Actb) ΔCt values for CFB −/− versus WT mice exposed to room air (fold difference). Candidates were examined from a number of categories including photoreceptor cell function (Rho, Opn1sw, Opn1mw, Rpe65), complement activation (C3, Cfb, Cfd, Cfh, Cd55, Cd59a), control of angiogenesis (Vegfa, Serpinf1), oxidative stress (Hif1a, Cp), autophagy (Lyz1, Lamp2, Klc3), and mitochondrial function (Mfn1, mt-Co1, Dnm1l, Ndufb8, Pfkfb1, Hmox1). Significant differences were identified in all six categories for CFB −/− mice, suggesting increased cone function and reduced energy production compared to WT animals. Data are expressed as mean ±SEM (n = 3 per condition; *, P<0.005; **, P<0.001). (TIF) [file pone.0067894.s003.tif]
