# Supplementary material for: Alternative Complement Pathway Deficiency Ameliorates Chronic Smoke-Induced Functional and Morphological Ocular Injury
Source: PLoS One. 2013 Jun 25;8(6):e67894. doi: 10.1371/journal.pone.0067894 (PMC3692454; doi:10.1371/journal.pone.0067894)
Supplement: Text S1 — Detection of CFB and RD8 Genotypes by PCR. (DOCX) [file pone.0067894.s004.docx]

**Detection of CFB and RD8 Mutations by PCR**

PCR-ready DNA was prepared from mouse tail biopsy samples using HotSHOT alkaline lysis and neutralizing reagents [[1](#_ENREF_1)]. PCR amplification for each reaction was carried out using 15 µl of GoTaq green master mix (Promega, M7122), 10 µl of nuclease-free water, and 1 µl of each diluted primer per 5 µl of DNA template. PCR primers for complement factor B (CFB) deficiency detection were as follows: 5’-CCGAAGCATTCCTATCCTCC-3’, 5’-CAGATGGGCTGACCGCTTCC-3’, and 5’-CTAGTCTTGTCTGCTTTCTCC-3’ [[2](#_ENREF_2)]. Reactions for CFB were initially denatured at 95˚C for 5 minutes followed by 38 cycles at 95˚C for 60 seconds, 54˚C for 60 seconds, 72˚C for 60 seconds, and a final extension at 72˚C for 10 minutes. Amplified DNA samples for CFB mutation detection were run with an aliquot of GeneRuler 100 bp Plus DNA ladder (Fermentas, SM0323). Amplicon sizes for the CFB wild type and mutant allele is equal to 748 and 610 bp, respectively. Primer sequences for the detection of an RD8 mutation included *mCrb 1, mF1*: 5’-GTGAAGACAGCTACAGTTCTGATC-3’; *mCrb 1, mF2*: 5’-GCCCCTGTTTGCATGGAGGAAACTTGGAAGACAGCTACAGTTCTTCTG-3’; and *mCrb 1, mR*: 5’-GCCCCATTTGCACACTGATGAC-3’ [[3](#_ENREF_3)]. In order to get the best PCR amplification results of RD8 sequences, the *mF1* and *mR* primer amounts were doubled to compensate for the larger *mF2* primer. PCR reactions for RD8 were denatured at 94˚C for 5 minutes followed by 35 cycles at 94˚C for 30 seconds, 65˚C for 30 seconds, 72˚C for 30 seconds, and a final extension at 72˚C for 10 minutes. Amplified DNA samples for RD8 mutation detection were run against the 50 bp HyperLadderV (Bioline, BIO-33057). Amplicon sizes for the RD8 wild type and mutant allele is equal to 220 and 244 bp, respectively. Since the RD8 wild type and mutant amplification products have a similar molecular weight, primer set reactions were carried out separately for each DNA template. Amplified DNA samples and corresponding ladders were separated using a 1.5% agarose gel containing ethidium bromide and visualized under UV light.

1. Truett, G.E., et al., *Preparation of PCR-quality mouse genomic DNA with hot sodium hydroxide and tris (HotSHOT).* Biotechniques, 2000. **29**(1): p. 52, 54.

2. Kapadia, S.B., et al., *Murine gammaherpesvirus 68 encodes a functional regulator of complement activation.* J Virol, 1999. **73**(9): p. 7658-70.

3. Mattapallil, M.J., et al., *The Rd8 mutation of the Crb1 gene is present in vendor lines of C57BL/6N mice and embryonic stem cells, and confounds ocular induced mutant phenotypes.* Invest Ophthalmol Vis Sci, 2012. **53**(6): p. 2921-7.
